# *Pempheris gasparinii*, a new species of sweeper fish from Trindade Island, southwestern Atlantic (Teleostei, Pempheridae)

**DOI:** 10.3897/zookeys.561.7263

**Published:** 2016-02-08

**Authors:** Hudson T. Pinheiro, Giacomo Bernardi, Luiz A. Rocha

**Affiliations:** 1 Department of Ecology and Evolutionary Biology, University of California Santa Cruz, Santa Cruz, CA 95060 USA; 2 Ichthyology Section, California Academy of Sciences, San Francisco, CA 94118 USA

**Keywords:** Endemism, COI, Vitória-Trindade Chain, oceanic island, Brazil, reef fish

## Abstract

*Pempheris gasparinii* **sp. n.** is described from five specimens, 59.1–68.0 mm in standard length. It is only known to occur in the shallow reefs of Trindade Island, 1200 km east of the Brazilian coast, in the southwestern Atlantic. *Pempheris gasparinii* is the third recognized species of *Pempheris* in the Atlantic Ocean. This new species is morphologically similar to its close relative, *Pempheris poeyi*, differing by the number of lateral-line scales (51–54 in *Pempheris gasparinii* vs. 47–49 in *Pempheris poeyi*), scales below lateral line (10–11 vs. 9), circumpeduncular scales (11–12 vs. 13), head and caudal peduncle lengths (2.7–3.3 vs 3.5–4.0 in head length). Moreover, *Pempheris gasparinii* shows a 4% genetic divergence from *Pempheris poeyi* at the cytochrome oxidase I locus (COI), consistent with a lineage split at the beginning of the Pleistocene. This new species represents the 12^th^ endemic fish species from Trindade Island.

## Introduction

The genus *Pempheris* Cuvier contains 69 valid species (Randall and Victor 2015), with two species known from the Atlantic Ocean: *Pempheris poeyi* Bean 1885 and *Pempheris schomburgkii* Müller & Troschel, 1848. *Pempheris* species generally display similar body shapes, however, these two species strongly differ by coloration (*Pempheris poeyi* is silvery while *Pempheris schomburgkii* is yellowish) and number of anal-fin rays (22–24 in *Pempheris poeyi* vs. 31–34 in *Pempheris schomburgkii*). *Pempheris schomburgkii* is widely distributed in the western Atlantic, from Bermuda (32°N) to Santa Catarina, Brazil (29°S). In contrast, a disjunct distribution has been previously assigned to *Pempheris poeyi*, with populations occurring in the Greater Caribbean and in Trindade Island, Brazil (Pinheiro et al. 2015), at least 5,000 km apart.

*Pempheris poeyi* was described from Cuba in 1885 (Bean 1885), and since then few specimens have been collected from localities such as the Bahamas, Grenada, Trinidad and Tobago, and Venezuela. Specimens identified as *Pempheris poeyi* were found at Trindade Island in the early 20^th^ century (Miranda Ribeiro 1919), and rediscovered 90 years later by Pinheiro et al. (2009). There is overlap in counts of dorsal- and anal-fin rays between the Greater Caribbean and Trindade populations, a common observation among *Pempheris* species (Mooi and Randall 2014), which likely explains why the new Trindade species described herein was misidentified in two recent checklists (Simon et al. 2013, Pinheiro et al. 2015, Randall and Victor 2015). Recent publications revealed that there is an abundance of undescribed cryptic species in *Pempheris* (Koeda et al. 2013, Mooi and Randall 2014, Randall and Bineesh 2014, Randall and Victor 2014, Randall et al. 2014). In this study, the use of genetic tools allowed us to revisit the Trindade specimens and update their taxonomic status. Thus, herein we describe a third Atlantic Ocean *Pempheris* species, so far only known from Trindade Island, at the end of the Vitória-Trindade Chain, 1200 km off the Brazilian coast.

## Methods

All specimens were collected using hand nets. Counts were performed using a microscope, and morphological characters were measured to the nearest 0.1 mm following Mooi and Randall (2014). Morphometric and meristic data for the type series are presented in Table 1. In the description, meristic values for the holotype are provided first, followed by the range of counts of paratypes in parentheses. Type specimens were deposited in the fish collections of the Universidade Federal do Espírito Santo (CIUFES), California Academy of Sciences (CAS), and Universidade Estadual de Campinas (ZUEC).

**Table 1.** Counts and measurements of selected type specimens of *Pempheris gasparinii* sp. n. and comparative specimens of *Pempheris poeyi* and *Pempheris schomburgkii* (data range) as percentages of standard length. “Circumped” = circumcaudal peduncular.

	Holotype	Paratypes				*Pempheris poeyi*	*Pempheris schomburgkii*
	CIUFES 3127	CAS 238409	CAS 238410	CIUFES 2432	ZUEC PIS 11233		
LL Scales	54LR	51L,52R	53L,54R	53L,52R	52LR	47–49	56–59
Scales above LL	2	2	2	2	2	1	3
Scales below	11	11	10	11	10	8–9	15–17
Circumped scales	12	11	11	12	12	13	14–17
Gill rakers	25	24	24	23	25	24	24–25
Dorsal fin	IV 8	IV 8	IV 9	IV 8	IV 9	IV 7–8	IV 8
Anal fin	III 24	III 24	III 24	III 24	III 25	III 24–26	III 31–34
Pectoral fin	15	15	15	15	15	14–15	I 15
Pelvic rays	I 5	I 5	I 5	I 5	I 5	I 5	I 5
SL (mm)	64.5	68.0	60.5	64.6	59.1	28.6–52.7	91.6–101.9
Body depth	36.5	37.1	37.2	37.3	36.9	36.2–41.0	41.4–48.4
Body width	14.1	13.5	15.0	14.2	15.2	14.6–17.1	15.0–16.6
Head length	31.5	27.2	30.6	31.4	31.3	32.1–36.1	26.3–33.3
Snout length	5.4	3.4	3.7	5.0	4.1	4.3–6.1	4.0–5.2
Orbit diameter	11.8	14.3	13.9	13.3	13.7	12.9–14.8	13.4–13.9
Interorbital width	12.6	12.7	9.8	9.4	8.5	8.4–11.2	8.5–9.3
Caudal-penducle depth	9.6	10.0	10.5	10.5	10.9	8.4–10.2	8.7–9.0
Caudal penducle length	7.1	10.1	8.1	7.7	9.1	6.6–9.7	7.9–8.5
Predorsal length	41.2	40.6	43.1	42.9	41.6	38.9–45.1	36.4–42.3
Pre-anal length	58.8	54.3	58.7	55.4	59.9	53.5–60.5	44.7–48.6
Prepelvic length	39.1	34.9	37.4	35.1	38.1	36.4–38.6	27.3–28.6
Base of dorsal fin	15.6	15.0	16.9	16.3	17.4	16.4–18.2	15.0–16.6
First dorsal spine	4.9	4.1	4.4	4.5	3.9	3.5–3.8	3.0–7.3
Fourth dorsal spine	17.1	16.5	21.7	18.2	17.7	16.6–18.9	18.5–20.9
Longest dorsal ray	22.0	19.5	22.3	23.1	21.8	17.6–21.9	20.3–23.3
Base of anal fin	35.0	34.9	35.0	34.3	37.6	33.5–42.6	47.2–47.8
First anal spine	intern	intern	intern	intern	intern	intern	1.9–2.7
Third anal spine	4.3	3.2	4.5	4.3	3.6	4.5–7.6	8.9–11.5
Longest anal ray	9.1	8.8	9.8	7.6	9.0	8.1–11.8	10.6–12.2
Caudal fin length	28.5	22.9	25.3	broken	broken	broken	25.4–29.6
Caudal concavity	17.1	17.1	17	broken	broken	broken	8.6–9.5
Pectoral-fin length	24.5	23.3	24.4	25.9	24.2	25.8–31.8	25.5–27.5
Pelvic-spine length	9.5	9.4	8.1	8.7	9.1	10.2–11.7	9.7–10.5
Pelvic fin length	14.9	13.2	13.7	13.0	14.0	13.1–18.5	10.5–13.6
Upper jaw length	16.1	18.4	16.8	17.5	18.8	15.9–18.4	15.3–17.4

Mitochondrial Cytochrome c oxidase subunit I (COI) DNA was analyzed for the new species. DNA extraction and PCR amplification of the COI were performed following Weigt et al. (2012). DNA sequences were then compared to those of the other two Atlantic *Pempheris* (*Pempheris poeyi* and *Pempheris schomburgkii*) downloaded from GenBank. Phylogenetic relationships were assessed by Maximum Likelihood (ML, GARLI software, Zwickl 2006), and Neighbor-Joining (MP, PAUP* software, Swofford 2003) methods. For Maximum Likelihood topologies, we conducted 10 independent runs in GARLI, using default settings and the automated stopping criterion, terminating the search when the ln score remained constant for 20,000 consecutive generations. Statistical confidence in nodes was evaluated using 1,000 non-parametric bootstrap replicates (Felsenstein 1985) for the NJ method, and 100 replicates for ML in GARLI, using the automated stopping criterion set at 10,000 generations. *Parapriacanthus* is the only other genus of Pempheridae and was used as an outgroup as in Azzurro et al. (2015) (sequence from GenBank, accession number JF494087). *Pempheris* sequences are from Azzurro et al. (2015), except for *Pempheris poeyi*, which is from this study. GenBank access numbers are KJ609406 (*Pempheris gasparinii* sp. n.), KT634057 (*Pempheris poeyi*), and KJ609388.1 and KJ609389.1 (*Pempheris schomburgkii*).

## Results

### Pempheris gasparinii sp. n.

Taxon classificationAnimaliaPerciformesPempheridae

http://zoobank.org/E4965E3A-F4BB-44DF-AE9B-51135B822B41

**Figure 1.**
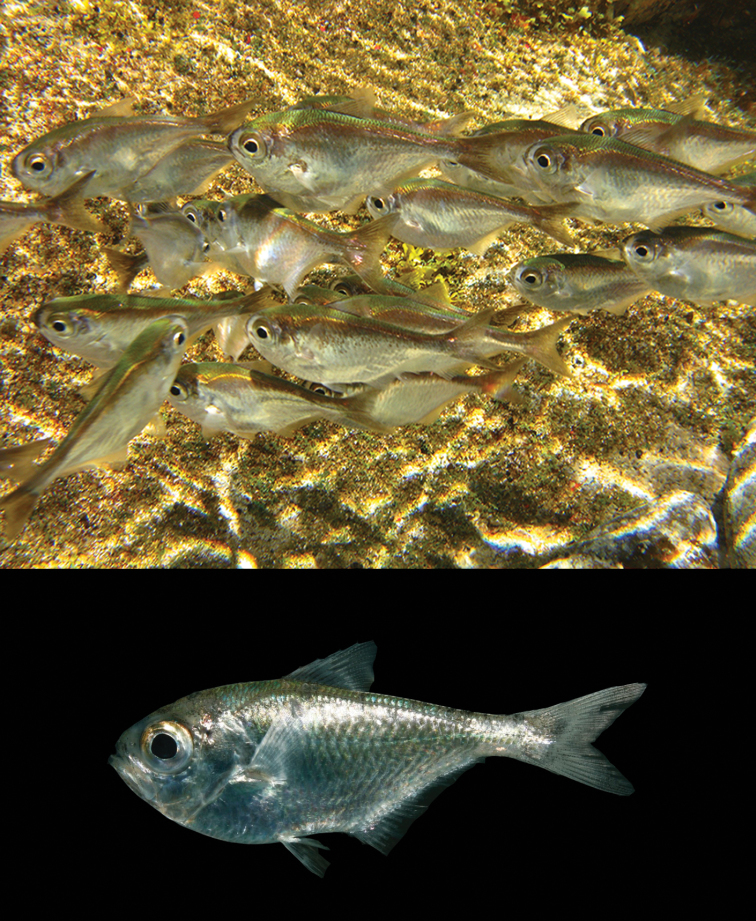
Underwater picture of *Pempheris gasparinii* sp. n. at the Parcel Pool, Trindade Island (top; photo J.L. Gasparini), and a specimen shortly after death (bottom; photo T. Simon).

**Figure 2.**
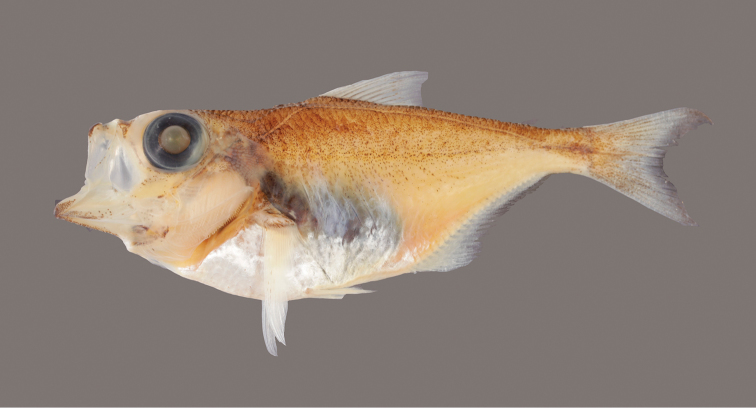
*Pempheris gasparinii* sp. n., preserved holotype, 64.5 mm SL, CIUFES 3127 (photo A. Barber).

Figs 1 , 2, Trindade sweeper

Pempheris poeyi : Miranda-Ribeiro 1919: 173
Pempheris poeyi : Pinheiro et al. 2009: 49
Pempheris poeyi : Simon et al. 2013: 2121
Pempheris poeyi : Pinheiro et al. 2015: 20

#### Type locality.

Trindade Island, Espírito Santo State, Brazil.

#### Holotype.

CIUFES 2432, 64.5 mm SL, GenBank KJ609406, Parcel pool, Trindade Island, Espírito Santo, Brazil. 20°30'S, 29°20'W, depth 1 m, collected by HT Pinheiro and JL Gasparini, 25 Jun 2009 (Figure 2).

#### Paratypes.

CAS 238409, 68.0 mm SL, CAS 238410, 60.5 mm SL, CIUFES 2432, 64.6 mm SL, ZUEC PIS 11233, 59.1 SL. Same data as holotype.

#### Comparative material.

*Pempheris poeyi*: CAS 238411 (one specimen, 52.7 mm SL, Curaçao), USNM 318952 (two specimens, 34.6 and 39.1 mm SL, Tobago), USNM 354575 (two specimens, 28.6 and 29.0 mm SL, Tobago); *Pempheris schomburgkii*: CAS 1595 (one specimen, 92.7 mm SL, Bahia, Brazil), CAS 32060 (two specimens, 48.7 and 39.7 mm SL, Barbados), CAS 236600 (one specimen, 99.2 mm SL, Curaçao), CAS 236604 (one specimen, 103.4 mm SL, Curaçao).

#### Diagnosis.

*Pempheris gasparinii* differs from its congeners by the following combination of character states: Head 3.2–3.7 in SL; body depth 2.7 in SL; head length 3.2–3.7 in SL; orbit diameter 1.9–2.7 in HL; caudal-peduncle depth 2.7–3.3 in HL; dorsal rays IV, 8–9; anal-fin rays III, 24–25; pectoral rays 15; lateral-line scales 51–54; scales below the lateral line 10–11; circumpeduncular scales 11–12; and gill rakers 23–25. Color in life mostly silvery, darker from mid-body to the lateral line and greenish above; fins are translucent with a darker tail. Color in alcohol light brown to silvery with darker dorsum and translucent fins; caudal fin darker. Additionally, mitochondrial DNA COI sequences show a divergence of at least 4% from all Atlantic congeners.

#### Description.

Dorsal-fin rays IV, 8 (IV, 8–9), all segmented rays branched; anal rays III, 24 (III, 24–25), first internal, all segmented rays branched; pectoral rays 15, the first rudimentary, second unbranched, remaining rays branched; pelvic rays I V (I V); principal caudal rays 9+9 (9–10+9), the median 16 branched (16–17); upper+lower procurrent caudal rays 6+6 (6–8+5–6), the most posterior of each side segmented distally; lateral-line scales 54 (51–53) to the base of the caudal fin, pored scales continuing to the end of the fin; gill rakers 6+19 = 25 (4–6+19–21).

Body moderately deep and compressed. Depth 2.7 (2.7) in SL and width 2.6 (2.4–2.7) in body depth; head length 3.2 (3.2–3.7) in SL and dorsal profile of head moderately convex; snout very short, 5.8 (6.3–8.2) in HL; orbit diameter 2.7 (1.9–2.4) in HL; interorbital width 2.5 (2.1–3.7) in HL; caudal-peduncle depth 3.3 (2.7–3.1) in HL; caudal-peduncle length 4.5 (2.7–4.1) in HL.

Mouth oblique, forming ~60°angle to horizontal axis of body; lower jaw slightly protruding when mouth fully closed; maxilla expanding posteriorly to a width two-thirds pupil diameter; upper jaw teeth very small, sharp, incurved, in two irregular rows anteriorly, narrowing to a single row posteriorly; lower jaw teeth small, in a patch with three to four irregular rows, outer one or two rows sharply nodular, inner two rows strongly recurved and narrowly sharp; vomer with expanded V-shaped patch (three to four irregular rows) of very small nodular teeth; palatines with a long and narrow patch (two irregular rows anteriorly to one posteriorly) of very small and medially curved teeth; tongue diamond shaped, upper surface with small papillae.

Gill rakers long, longest gill filaments two-thirds the length of longest gill raker. Gill opening extending dorsally, near level of center of orbit. Margin of preopercle smooth. Anterior and posterior nostril apertures vertically oval, positioned in front of dorsal edge of pupil, and separated by narrow septum; no membranous flap on nostril edges. Most scales cycloid, except finely ctenoid posteriorly in interorbital area, on nape, on dorsal area posteriorly to dorsal fin, and above lateral line and chest. Small scales covering slightly more than basal half of anal fin and slightly less than half of caudal fin.

Origin of dorsal fin posterior to vertical alignment of rear base of pectoral fin; predorsal length 2.4 (2.3–2.5) in SL; dorsal-fin base 6.4 (5.7–6.7) in SL; first dorsal spine short, 6.4 (6.6–8.0) in HL; fourth dorsal spine longest, 1.8 (1.4–1.8) in HL; first or second dorsal segmented rays longest, 1.4 (1.4) in HL; origin of anal fin aligned with end of dorsal fin base, preanal length 1.7 (1.7–1.8) in SL; anal-fin base short, 2.9 (2.7–2.9) in SL; first anal spine internal and very short; third anal spine short, 7.3 (6.7–8.8) in HL; longest anal segmented ray 3.4 (3.1–3.5) in HL; caudal-fin length 3.5 (4.0–4.4) in SL; caudal concavity 1.8 (1.6–1.8) in HL; second or third branched pectoral ray usually longest, 4.1 (3.9–4.3) in SL; origin of pelvic fins slightly posterior to rear base of pectoral fins, prepelvic length 2.6 (2.6–2.9) in SL; pelvic spine 3.3 (2.9–3.8) in HL; pelvic-fin length 2.1 (2.1–2.4) in HL.

#### Color in life.

Body mostly silvery, darker from mid-body to lateral line and greenish above; fins translucent with a darker tail (Figure 1).

#### Color in alcohol.

Body light brown, darker dorsally; fins pale except caudal fin with basal melanophores (Figure 2).

#### Etymology.

The specific name honors our ichthyologist colleague and friend João Luiz Rosetti Gasparini, one of the pioneers on the study of taxonomy and biodiversity of reef fishes in Brazil and Trindade Island. “Gaspa” has contributed to nearly half of the descriptions of reef-fish species from Brazilian waters in the last two decades. To be treated as a noun in apposition.

#### Distribution and habitat.

**Figure 3.**
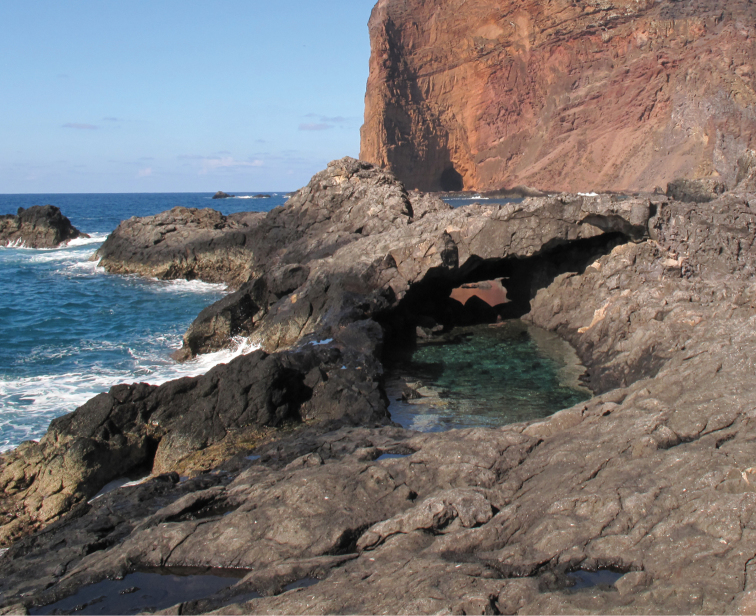
Parcel Pool, Trindade Island, Brazil, type locality of *Pempheris gasparinii* sp. n. (photo H.T. Pinheiro).

*Pempheris gasparinii* sp. n. is known only from the type locality, Trindade Island, Espírito Santo, Brazil. It has only been found schooling in the very shallow waters of the rocky Parcel pools (Figure 3).

#### Genetic data.

**Figure 4.**
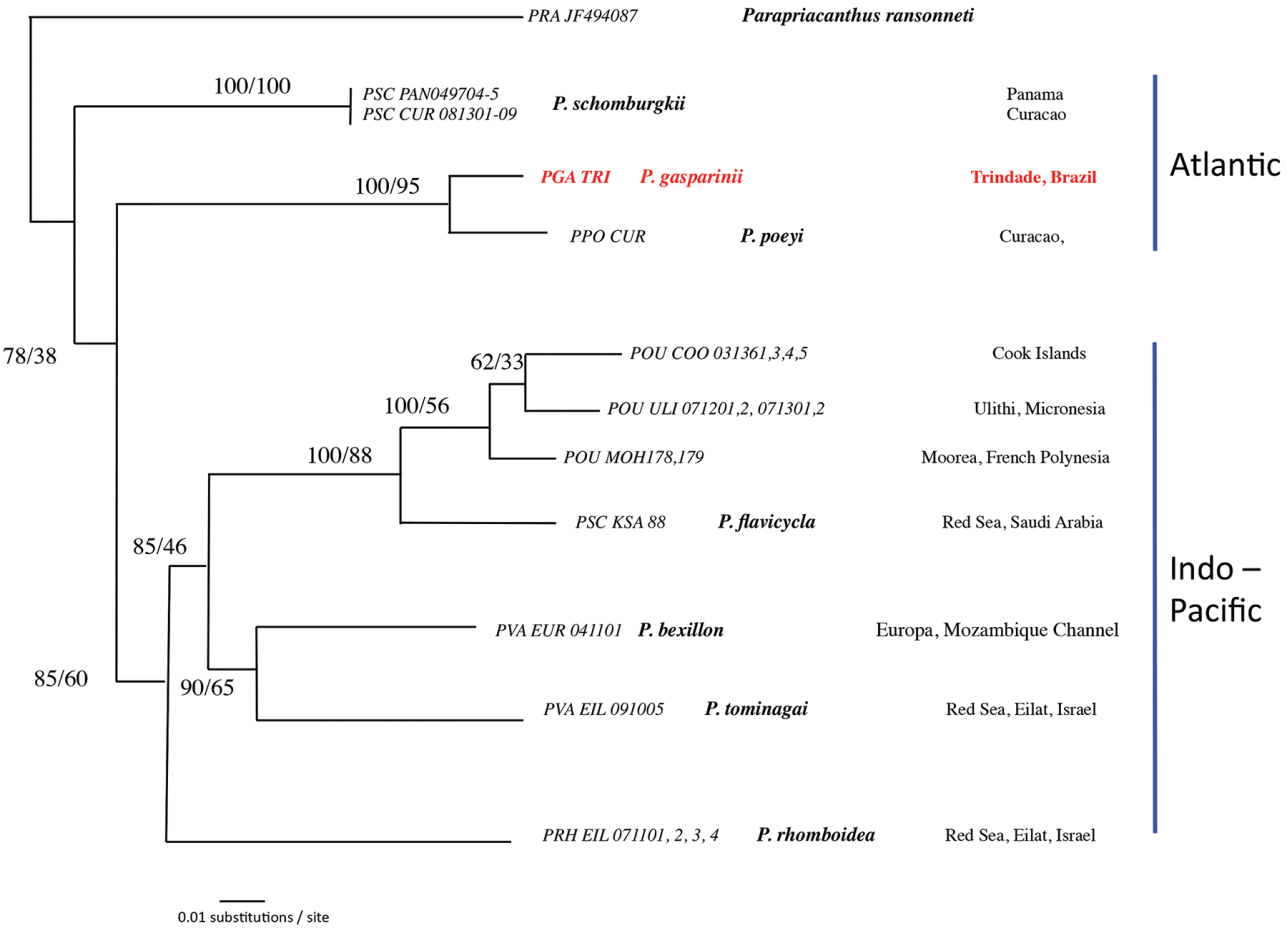
Maximum Likelihood tree of Atlantic *Pempheris* based on the mitochondrial cytochrome oxidase 1 (CO1) marker. Labels correspond to sample names (following Azzurro et al. 2015), species names (when identified), collection locality, and biogeographic region. Bootstrap support (1000 replicates) for Neighbor-Joining and Maximum Likelihood are given at the node, in this order. The new species, *Pempheris gasparinii*, is labeled in red.

A maximum likelihood phylogenetic reconstruction of Atlantic *Pempheris*, based on the mitochondrial cytochrome oxidase 1 (CO1) marker, is presented in Figure 4. The Kimura-2 genetic distance between *Pempheris gasparinii* sp. n. and its closest relative (*Pempheris poeyi*) is 4%.

#### Comparative remarks.

*Pempheris gasparinii* differs from its Atlantic Ocean congeners by the smaller number of soft anal-fin rays (24–25) compared to *Pempheris schomburgkii* (31–34), and greater number of lateral-line scales (51–54) and scales below the lateral line (10–11) compared to *Pempheris poeyi* (47–49, and 9 scales respectively). *Pempheris gasparinii* also has fewer circumpeduncular scales (11–12 vs 13), a smaller head (3.2–3.7 vs 2.8–3.1 in SL) and higher caudal peduncle depth (2.7–3.3 vs 3.5–4.0 in head length) compared to *Pempheris poeyi*.

## Discussion

As stated by Mooi and Randall (2014), assignments of original or new names to *Pempheris* species is remarkably difficult because: 1) the poor condition and descriptions of type material; and 2) traditional meristics (e.g. fin ray counts) are not very informative at the species level due either to too little or too much character variability. For instance, because of its similarity to *Pempheris poeyi*, *Pempheris gasparinii* is misidentified in the most recent biodiversity checklists of Trindade Island (Pinheiro et al. 2009, 2015, Simon et al. 2013).

A phylogenetic tree of *Pempheris* of the Atlantic based on COI placed *Pempheris poeyi* and *Pempheris gasparinii* as sister species (Figure 4). The two species show a 4% sequence divergence at this locus, which is consistent with a speciation process initiated in the beginning of the Pleistocene. With only one sequence from each of the two species and thus no information on intraspecific genetic divergences, we cannot definitively use the genetic data to separate the two species. However, assuming that intraspecific divergence is low, as it is in *Pempheris schomburgkii* from Panama and Curacao (Fig. 4), the combined genetic and morphological data easily distinguish the two species. The disjunct distribution associated with the genetic divergence of the species suggests that *Pempheris gasparinii* might be a relict species. Similarly to other recently described species from Trindade Island (Pinheiro et al. 2010, Rocha et al. 2010), *Pempheris gasparinii* seems to have been preserved in the Vitória-Trindade Chain while the Pleistocene sea-level changes extirpated the lineages along the adjacent Brazilian coast. This species represents the 12^th^ endemic fish from Trindade Island. Thus, following the diversity reported by Pinheiro et al. (2015) and criteria of reef fishes established by Floeter et al. (2008), the proportion of endemics found in the Vitória-Trindade Chain (191 species) and Trindade Island (137 species) today is 6% and 9% respectively.

## Supplementary Material

XML Treatment for Pempheris gasparinii
